# Two mechanisms of nanoparticle generation in picosecond laser ablation in liquids: the origin of the bimodal size distribution†
†Electronic supplementary information (ESI) available. See DOI: 10.1039/c7nr08614h


**DOI:** 10.1039/c7nr08614h

**Published:** 2018-03-08

**Authors:** Cheng-Yu Shih, René Streubel, Johannes Heberle, Alexander Letzel, Maxim V. Shugaev, Chengping Wu, Michael Schmidt, Bilal Gökce, Stephan Barcikowski, Leonid V. Zhigilei

**Affiliations:** a Department of Materials Science and Engineering , University of Virginia , 395 McCormick Road , Charlottesville , Virginia 22904-4745 , USA . Email: lz2n@virginia.edu; b Technical Chemistry I and Center for Nanointegration Duisburg-Essen (CENIDE) , University of Duisburg-Essen , Universitaetsstr. 7 , Essen 45141 , Germany . Email: bilal.goekce@uni-due.de; c Institute of Photonic Technologies , Friedrich-Alexander University Erlangen-Nürnberg , Konrad-Zuse-Straße 3/5 , Erlangen 91052 , Germany

## Abstract

The synthesis of chemically clean and environmentally friendly nanoparticles through pulsed laser ablation in liquids has shown a number of advantages over conventional chemical synthesis methods and has evolved into a thriving research field attracting laboratory and industrial applications. The fundamental understanding of processes leading to the nanoparticle generation, however, still remains elusive. In particular, the origin of bimodal nanoparticle size distributions in femto- and picosecond laser ablation in liquids, where small nanoparticles (several nanometers) with narrow size distribution are commonly observed to coexist with larger (tens to hundreds of nanometers) ones, has not been explained so far. In this paper, joint computational and experimental efforts are applied to understand the mechanisms of nanoparticle formation in picosecond laser ablation in liquids and to explain the bimodal nanoparticle size distributions. The results of a large-scale atomistic simulation reveal the critical role of the dynamic interaction between the ablation plume and the liquid environment, leading to the generation of large nanoparticles through a sequence of hydrodynamic instabilities at the plume-liquid interface and a concurrent nucleation and growth of small nanoparticles in an expanding metal-liquid mixing region. The computational predictions are supported by a series of stroboscopic videography experiments showing the emergence of small satellite bubbles surrounding the main cavitation bubble generated in single pulse experiments. Carefully timed double pulse irradiation triggers expansion of secondary cavitation bubbles indicating, in accord with the simulation results, the presence of localized sites of laser energy deposition (possibly large nanoparticles) injected into the liquid at the early stage of the bubble formation.

## Abstract 2

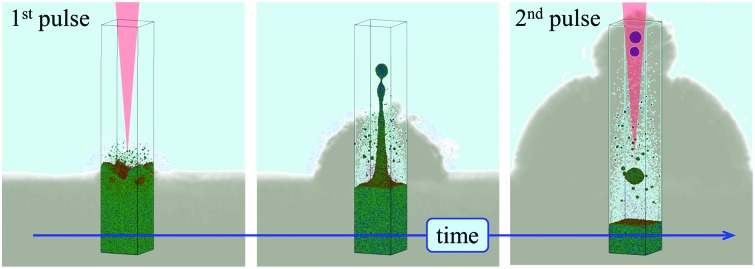
Novel mechanisms of nanoparticle generation in laser ablation in liquids are revealed in atomistic simulations and verified in experiments.

## 

The production of clean colloidal solutions of nanoparticles through laser ablation in liquids (LAL) has evolved over the last decade into a mature research field with a large and growing number of practical applications.^1^–^4^ While the challenges of increasing productivity and broadening the range of materials available for nanoparticle generation are successfully addressed in the ongoing exploration of the space of experimental parameters,^3^,^5^ the goal of achieving a narrow nanoparticle size distribution by direct one-step LAL still remains elusive. In particular, the broad size distributions, where the desired small nanoparticles coexist with larger ones (tens to hundreds of nanometers), are commonly observed in LAL experiments regardless of the pulse durations. However, in experiments performed with short (<100 ps) laser pulses, bimodal nanoparticle size distribution^6^–^9^ becomes apparent, particularly when the nanoparticle statistic is sufficient, and experimental setup does not facilitate nanoparticle fragmentation through post-irradiation (*i.e.*, the flow chamber design and appropriate laser repetition rate prevent interaction of laser pulses with already generated nanoparticles).

The bimodal size distribution in the colloidal solutions produced by LAL presents an obstacle for direct use of the colloids in a number of advanced photonic, catalytic, and biomedical applications,^1^–^4^,^10^–^12^ where a narrow monomodal nanoparticle size distribution is required. In catalysis based on Pt group nanoparticles, for example, the large nanoparticles may account for a major fraction of the total mass of the catalyst but make little contribution to the catalytic activity, which is controlled by the mass-specific surface area and is dominated by the small nanoparticles.^13^ Biomedical applications, such as Alzheimer disease research, is another area where the fine control over nanoparticle sizes is critical. The laser-generated nanoparticles are advantageous as the ligand grafting density can easily be set,^14^ but precise control over ligand-to-nanoparticle ratio and nanoparticle dose requires a narrow monomodal nanoparticle size distribution. Hence, additional steps such as centrifugation,^15^ salinity size quenching,^16^ or other types of post-processing have to be performed on laser-synthesized colloids^3^ to obtain a desirable monomodal nanoparticle size distribution.

To fully utilize the potential of pulsed laser ablation in liquids for generation of nanoparticles with well-controlled structure, composition, and size distribution, one needs to improve the understanding of the laser-induced processes responsible for the generation of colloidal nanoparticles. Such understanding can only emerge from simultaneous progress in time-resolved experimental probing, theoretical description, and computational modeling of laser-induced processes.

The experimental data on the nanoparticle generation in LAL is mostly indirect and is based on analysis of the dynamics of cavitation bubble generated due to the interaction of the ablation plume with liquid environment. For nanosecond laser ablation in liquids, the cavitation bubble dynamics has been explored through various optical techniques, including light scattering,^17^ shadowgraphy,^18^,^19^ and stroboscopic videography.^20^ The understanding of connections between the nanoparticle generation mechanisms and cavitation bubble dynamics has recently been greatly advanced by the results of small angle X-ray scattering (SAXS) probing of the evolution of the nanoparticle size distribution with respect to time and position inside the cavitation bubble generated in nanosecond LAL.^16^,^21^–^23^ The experimental evidence suggests that cavitation bubble serves as a reaction chamber for the nanoparticle nucleation, growth, coalescence, and solidification, whilst two or more distinct nanoparticle populations may appear at different stages of the bubble expansion and collapse. While the SAXS results provide first-hand quantitative insights into the nanoparticle generation in nanosecond LAL, these insights cannot be readily extrapolated to picosecond or femtosecond LAL, where the processes responsible for the nanoparticle generation are likely to be distinct from those in the nanosecond irradiation regime. Moreover, the initial and perhaps most critical stage of the nanoparticle formation at the onset of the bubble generation and expansion still remains beyond the temporal and spatial resolution of any of the experimental techniques.

The theoretical and computational treatments of laser-material interactions in liquids have a potential to complement the experimental efforts but have been hampered by the highly non-equilibrium nature of the laser-induced processes. The continuum-level modeling, in particular, while successful in providing initial insights into the effect of the spatial confinement on the ablation plume expansion and phase decomposition,^24^,^25^ has been suffering from the lack of an adequate description of some of the key processes, such as vaporization of the liquid, mixing of the ablation plume with liquid environment, and generation of nanoparticles in the mixing region. The atomic-level molecular dynamics (MD) computational technique is suitable for exploring fast non-equilibrium phenomena and has been actively used for simulation of laser-materials interactions in vacuum, as reviewed in ref. 26–28. The high computational cost of atomistic treatment of both the irradiated target and the liquid environment, however, has been hampering the extension of the domain of applicability of the MD technique to LAL. New opportunities in this area have been provided by recent development of a computationally efficient coarse-grained representation of liquid environment^29^–^31^ and advanced boundary conditions,^32^ which led to the design of a combined atomistic – coarse-grained MD model capable of revealing the specific characteristics of laser-material interactions in liquids.^29^,^33^,^34^

In this paper, we report the results of a computational study supported by experimental observation aimed at revealing the mechanisms of nanoparticle formation in picosecond LAL and explaining the origin of the bimodal nanoparticle size distributions. A large-scale MD simulation performed for a Ag target irradiated by a picosecond laser pulse in water provides important insights into the initial stage of the ablation plume formation and interaction with water environment, and predicts the existence of two distinct mechanisms of the nanoparticle formation, namely, the nucleation and growth of small nanoparticles in the metal-water mixing region and the formation of larger nanoparticles through the breakup of the superheated molten metal layer generated at the plume-water interface. The latter mechanism, involving injection of the large nanoparticles into liquid above the emerging bubble, is further supported by the cavitation bubble imaging experiments, where small satellite bubbles surrounding the main cavitation bubble are observed upon single picosecond pulse irradiation, and the activation of the expansion of secondary bubbles upon properly timed double pulse irradiation is demonstrated.

## Results and discussion

### Computational prediction of two nanoparticle generation mechanisms

**Fig. 1:**
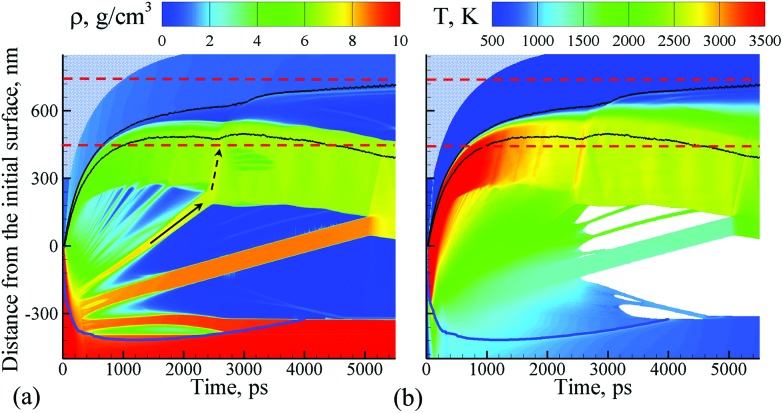
Density (a) and temperature (b) contour plots predicted in atomistic simulation of laser ablation of a bulk silver target irradiated in water by a 10 ps laser pulse at an absorbed fluence of 600 mJ cm^–2^. The blue line shows the location of the melting and solidification fronts. The two black lines outline the water-Ag mixing region defined as a region where both water molecules and Ag atoms are present. The blue dot background represents the presence of water beyond the pressure-transmitting boundary applied at the top of the water layer that is explicitly simulated with coarse-grained MD. The two dashed red lines outline the region for which snapshots of atomic configurations are shown in Fig. 2. The solid and dashed arrows show the trajectory of a spalled layer and a pressure pulse generated by collision of this layer with the molten metal layer accumulated at the interface with water environment, respectively.

**Fig. 2:**
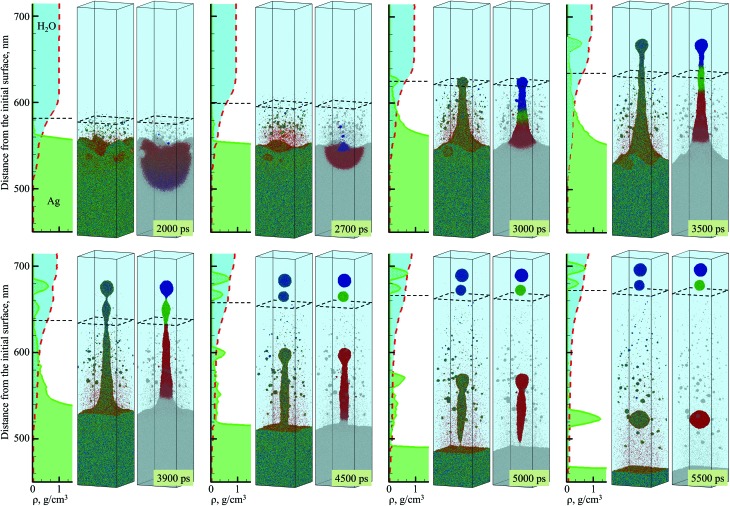
Snapshots of atomic configurations and density distribution predicted in atomistic simulation of laser ablation of a bulk silver target irradiated in water by a 10 ps laser pulse at an absorbed fluence of 600 mJ cm^–2^. Only a part of the computational system from 450 to 715 nm with respect to the initial surface of the silver target is shown in the figure. Two representations of atomic configurations are provided for each moment of time. On the left side of the paired snapshots, the atoms are colored according to their potential energies, from blue for the crystalline nanoparticles, to green for molten Ag, and to red for individual Ag atoms. On the right side of the paired snapshots, the atoms are colored based on IDs of three nanoparticles generated through the rupture of the liquid nanojet (each color except grey corresponds to atoms that end up in one of the three nanoparticles). The molecules representing water environment are blanked and the presence of water is illustrated schematically as a bright blue region above the Ag target. The degree of water-silver mixing is illustrated by density plots shown as functions of distance from the substrate for both water and silver to the left from the corresponding snapshots; the red dashed line and light blue fill color represent water density distribution, the green solid line and light green fill color represent Ag density distribution. The black dashed squares in the atomistic snapshots and the horizontal dashed lines in the density plots show approximate positions of the diffuse “boundary” between the dense water and low-density mixing region defined here as the position where the water density is 0.6 g cm^–3^.

**Fig. 3:**
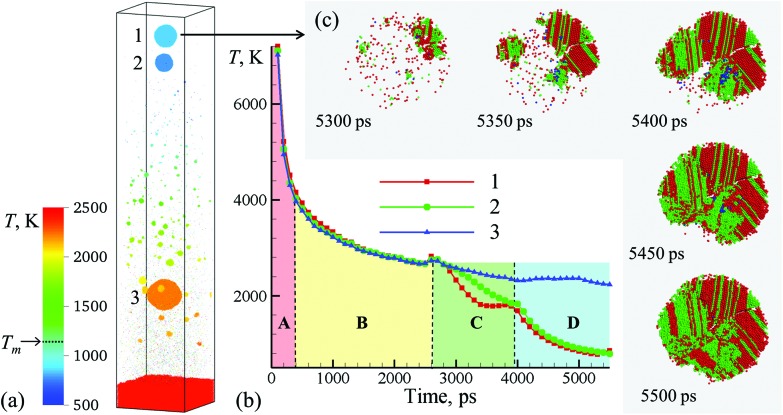
(a) Snapshot of the final configurations obtained for 5.5 ns after the laser pulse in a simulation of a bulk Ag target irradiated in water by a 10 ps laser pulse at an absorbed fluence of 600 mJ cm^–2^. Only a part of the computational system from 450 to 715 nm with respect to the initial surface of the Ag target is shown in the snapshot. The atoms in the snapshot are colored by local temperature. (b) The time dependence of the average temperature of atoms that belong to the one of the three nanoparticles generated through the rupture of the liquid nanojet shown in Fig. 2. (c) The process of crystallization in the topmost nanoparticle (15 nm in diameter) ejected from the liquid nanojet. The atoms are colored according to their local structural environment, so that the fcc, hcp, and bcc atoms are colored green, red, and blue, respectively, while the atoms that belong to the melted parts of the nanoparticles, crystal defects, and free surfaces are blanked.

The simulation, illustrated in Fig. 1–3, is performed for a laser pulse duration of 10 ps and an absorbed laser fluence of 600 mJ cm^–2^, which is about three times above the threshold fluence for the transition from spallation to phase explosion regimes of laser ablation of Ag in vacuum.^35^,^36^ In the phase explosion regime, the main driving force responsible for the material ejection is the rapid release of vapor in a strongly superheated surface region of the target.^37^ In vacuum, the explosive release of vapor drives the decomposition of the superheated region of the target into vapor, atomic clusters, and small droplets. Deeper into the target, the propagation of unloading wave generated due to the expansion of the top part of the target induces cavitation in the molten material and may result in the ejection of larger droplets through a process commonly referred to as photomechanical spallation.^38^–^40^ The presence of liquid environment, however, drastically alters the ablation dynamics. All the material, which in vacuum would freely expand away from the target as a mixture of small liquid droplets and vapor, is now confined by water and collected at the plume-water interface into a dense hot molten layer, as shown in Fig. 1.

The molten layer rapidly grows in thickness and cools down as more Ag droplets originating from deeper and colder parts of the target join it. At the same time, water in contact with the hot metal layer is brought into supercritical state and starts to expand, exerting additional downward pressure on the metal layer. The interaction with the water overlayer results in a rapid deceleration of the layer, with magnitude of the deceleration being as high as ∼6.3 × 10^12^ m s^–2^ at the initial stage of the plume-water interaction, at 100 ps, and decreasing down to ∼7.4 × 10^11^ m s^–2^ by 500 ps. This rapid deceleration directed from the lighter supercritical water to the higher density metal layer creates conditions for the development of the nanoscale Rayleigh-Taylor instability^41^,^42^ at the decelerated interface. The results of earlier MD simulations combined with quantitative analysis of the fastest growing wavelength and the characteristic time of the exponential growth of small perturbations in the Rayleigh-Taylor instability generated at comparable levels of interface acceleration^33^ (or equivalent gravitational field^43^,^44^) predict the emergence of the nanoscale interface morphology on the timescale of hundreds of picoseconds. These predictions are consistent with the results of present simulation, where the initial nanoscale roughness of the metal-water interface emerges within the first ∼500 ps after the laser pulse (see Supplementary Fig. S3†) and evolves into a deep trough (single finger of the Rayleigh-Taylor instability) by the time of 2000 ps, as can be seen in the first snapshot shown in Fig. 2.

The roughening of the interface between the hot metal layer and the supercritical water is also reflected in the expansion of the metal-water mixing region that is outlined by two black lines in Fig. 1. The mixing proceeds not only by active evaporation of Ag atoms into the low-density supercritical water region that serves as a precursor of the cavitation bubble observed in LAL experiments, but also by the penetration of water into the metal layer roughened by the Rayleigh–Taylor instability. The roughening of the metal-water interface combined with the general limited stability of thin liquid films^45^,^46^ may result in eventual partial or complete disintegration of the metal layer leading to the generation of large nanoparticles in the lower part of the low-density metal-water region, as has been observed in a recent simulation of laser ablation of a thin Ag film in water.^29^ The results of the present simulation, however, suggest an alternative scenario in which the large nanoparticles can be directly injected into the high-density colder water region located *above* the low-density mixing region. This scenario is illustrated in Fig. 2 and is described next.

As mentioned above, the hot molten metal layer formed at the interface with the water environment is growing through the addition of new metal droplets or layers joining it from below. As time progresses, these droplets/layers become larger and colder, as they originate from deeper regions of the target and are ejected with the assistance of photomechanical processes. The backside impact of the material joining the hot molten metal layer can induce pressure pulses in the layer that are sufficiently strong to interfere with rough metal-water interface and result in the emission of metal nanojets into the low-density mixing region. As an example of the sequence of processes leading to the nanojetting, we can consider the collision of a spalled layer ejected from a relatively deep part of the target (the trajectory of this layer is marked by a solid arrow in Fig. 1a) with the molten metal layer accumulated at the metal-water interface. The collision occurs at ∼2600 ps and produces a pressure pulse (marked by the dashed arrow in Fig. 1a) that can be identified from the transient densification of the molten metal layer (Fig. 1a) and the corresponding temperature spike due to the rapid adiabatic compression^47^ (Fig. 1b). The interaction of the pressure pulse with the metal-water interface roughened due to the Rayleigh-Taylor instability leads to the emission of a nanojet that rapidly emerges from the metal layer between 2.7 and 3 ns, and disintegrates into three large nanoparticles with diameters of 12 nm, 15 nm, and 19 nm by the time of 5 ns, Fig. 2.

The origin of atoms that end up in each of the three nanoparticles is shown in the right frames of the pairs of snapshots shown in Fig. 2. As can be seen from these snapshots, the atoms that contribute to the large nanoparticles are mostly located within the trough region of the molten metal layer before the backside impact, suggesting that the roughness of the interface plays an essential role in the formation of the nanojet. Indeed, the dynamics of material redistribution from the trough of the interface to the nanojet is consistent with conclusions of theoretical analysis of the Richtmyer-Meshkov instability produced when a shock wave impinges a roughened interface between materials of different density.^48^,^49^

The formation and subsequent rupturing of the nanojet not only produces three large nanoparticles but also launches two of them past the low-density mixing region directly into dense and relatively cold water environment. The boundary between the low-density mixing region and dense water environment is defined at water density of 0.6 g cm^–3^ and marked in the atomistic snapshots shown in Fig. 2 by black dashed squares. The process of jetting that crosses the boundary at ∼3000 ps can be seen in Fig. 2 from both the snapshots and the water and Ag density profiles shown next to the corresponding snapshots. Two of the green peaks that correspond to the Ag nanoparticles in the density profiles appear in the region where the water density is comparable to its liquid state density, and the metal atoms and small clusters produced through evaporation from the hot metal layer are absent. These two nanoparticles do not have net velocity with respect to water and can be expected to move along with the surrounding water as the low-density mixing region expands and evolves into a cavitation bubble.

The injection of large liquid droplets into the dense water environment makes a strong impact on their cooling rate and solidification. The thermal history of material contributing to the three nanoparticles produced from the disintegration of the nanojet is shown in Fig. 3b, where four stages of cooling can be distinguished. The initial sharp temperature drop from the level exceeding the critical temperature of Ag (stage marked as **A** in Fig. 3b) corresponds to the explosive phase decomposition of the superheated surface region of the irradiated target into vapor and liquid. The material that experienced the phase decomposition is accumulated at the interface with the water environment, forms a hot molten layer that further cools down mostly due to the colder Ag originating from deeper parts of the target joining the layer (stage **B** in Fig. 3b). This stage continues until ∼2600 ps, when the pressure pulse generated by the impact from a spalled layer initiates the active hydrodynamic flow leading to the nanojet formation. Following the initial temperature spike related to the transient compression of the material, the nanojet is generated and the temperature starts to drop (stage **C** in Fig. 3b). At this stage, the temperature trajectories calculated by averaging over atoms contributing to the three droplets start to diverge, with the top part of the nanojet (colored blue and green in the right frames of the pairs of snapshots shown in Fig. 2) cooling faster due to more vigorous extension and interaction with colder water environment. The final stage **D** in Fig. 3b starts with disintegration of the nanojet into individual droplets. The disintegration itself leads to the surface energy minimization (and corresponding increase in the thermal energy) as the droplets attain spherical shape, which shows up as plateaus or even small transient increases in the temperature profiles. Following the separation from the nanojet, the droplets continue to cool due to the interaction with the surrounding water. At this stage, the thermal trajectories of the two droplets injected into dense water environment and the one left behind in the low-density precursor of the cavitation bubble sharply diverge. While the lowest nanoparticle located near the hot molten layer cools slowly and its temperature remains above 2000 K at the end of the simulation, the two upper nanoparticles experience an effective cooling rate of ∼7 × 10^11^ K s^–1^ during stage **D** of the simulation and reach temperature as low as 30% below the equilibrium melting temperature of Ag.

The rapid quenching to the conditions of deep undercooling triggers the onset of solidification in the topmost nanoparticle, which proceeds through the nucleation of several crystallites at ∼5250–5300 ps followed by their rapid growth and complete solidification of the nanoparticle within the following 200 ps, as shown in Fig. 3c. The structural analysis of the nanoparticle performed for different moments of time during the solidification reveals the transient appearance of small domains of body centered cubic (bcc) structure (blue atoms that can be seen between 5350 and 5450 ps) as well as cross-nucleation of face centered cubic (fcc) and hexagonal close packed (hcp) regions with . The structural analysis of the nanoparticle performed for different moments of time during the solidification reveals the transient appearance of small domains of body centered cubic (bcc) structure (blue atoms that can be seen between 5350 and 5450 ps) as well as cross-nucleation of face centered cubic (fcc) and hexagonal close packed (hcp) regions with 〈111〉fcc//〈0001〉hcp orientation relationship (green and red atoms in 111. The structural analysis of the nanoparticle performed for different moments of time during the solidification reveals the transient appearance of small domains of body centered cubic (bcc) structure (blue atoms that can be seen between 5350 and 5450 ps) as well as cross-nucleation of face centered cubic (fcc) and hexagonal close packed (hcp) regions with 〈111〉fcc//〈0001〉hcp orientation relationship (green and red atoms in fcc//. The structural analysis of the nanoparticle performed for different moments of time during the solidification reveals the transient appearance of small domains of body centered cubic (bcc) structure (blue atoms that can be seen between 5350 and 5450 ps) as well as cross-nucleation of face centered cubic (fcc) and hexagonal close packed (hcp) regions with 〈111〉fcc//〈0001〉hcp orientation relationship (green and red atoms in 0001. The structural analysis of the nanoparticle performed for different moments of time during the solidification reveals the transient appearance of small domains of body centered cubic (bcc) structure (blue atoms that can be seen between 5350 and 5450 ps) as well as cross-nucleation of face centered cubic (fcc) and hexagonal close packed (hcp) regions with 〈111〉fcc//〈0001〉hcp orientation relationship (green and red atoms in hcp orientation relationship (green and red atoms in Fig. 3c). The resulting ultra-fine grained polycrystalline structure of the nanoparticle featuring multiple stacking faults, twin boundaries, and platelets of metastable hcp structure illustrates the possibility of the generation of nanoparticles with highly non-equilibrium metastable structures, defects,^12^ and phases under the conditions of extreme quenching rates that can be realized in LAL.^50^,^51^ Although the topmost nanoparticle (nanoparticle #1 in Fig. 3a) was the coldest one during most of the duration of stage **D** in Fig. 3b, the reheating due to the release of the latent heat of solidification brings its temperature above the second smaller nanoparticle (nanoparticle #2 in Fig. 3a) by the end of the simulation. Structural analysis of the second nanoparticle reveals the appearance of a small nucleus of the crystal phase at the end of the simulation, and one may expect that this nanoparticle would solidify within the following 100–200 ps if the simulation would be continued.

The third and largest nanoparticle generated through the nanojet disintegration (nanoparticle #3 in Fig. 3a) is located in the low-density part of the metal-water mixing region, where it coexists with numerous small nanoparticles generated through the nucleation and growth from Ag atoms that are continuously evaporating from the hot metal layer. As seen from Fig. 1b, the temperature in the mixing regions, while staying above the critical temperature of water, is close and, in the upper part, even below the melting temperature of Ag. As a result, the vapor Ag atoms rapidly condense forming small nanoparticles on a timescale of just several nanoseconds after the laser irradiation. The kinetics of the nanoparticle formation through the nucleation and growth in the mixing region is briefly discussed in ESI† and illustrated by Supplementary Fig. S4.† This nanoparticle generation mechanism has also been observed in recent atomistic simulations of laser ablation of Ag films and bulk targets in water,^29^,^33^ and is consistent with the results of time-resolved SAXS measurement^16^,^21^–^23^ suggesting that the “primary” particles with diameters less than 10 nm are likely to form through the condensation from the vapor phase at the initial stage of the cavitation bubble expansion.^21^

The computational prediction that larger nanoparticles, in the size range of tens of nanometers, can also be generated during the first nanoseconds after the laser irradiation, however, might not be directly associated with the whole fraction of so-called “secondary” particles identified in SAXS experiments.^16^,^21^–^23^ This secondary fraction is likely to consist of agglomerates and large spherical nanoparticles that cannot be differentiated *in situ*.^16^ Moreover, the SAXS experiments have been performed with nanosecond laser pulses, and the ablation process may proceed rather differently as compared to the picosecond ablation. In the nanosecond LAL, the secondary particles have been speculated to mostly form through collisions and agglomeration of primary particles,^21^ although the possibility of multiple pathways for generation of secondary particles have recently been considered as well.^16^ Indeed, first results of MD simulations performed with longer, sub-ns to ns, laser pulses (to be reported elsewhere) suggest that the generation of large nanoparticles through the formation and decomposition of a dense molten metal layer at the ablation plume-water environment interface is also activated in the nanosecond LAL.

Neither in the nanosecond LAL experiments nor simulations, however, the nanoparticles are detected beyond the cavitation bubble boundary. The computational prediction that, in the picosecond LAL, the large nanoparticles generated through the cascade of hydrodynamic instabilities can be directly ejected and embedded in the dense water region beyond the cavitation bubble boundary, marked schematically by dashed squares in Fig. 2, goes against the commonly accepted view that the nanoparticles are generated and confined within the cavitation bubble and are only released into liquid environment when the cavitation bubble collapses.^19^,^22^ At the same time, the computational prediction of the injection of large nanoparticles into the dense water environment suggests a unique feature of the picosecond LAL. In order to verify this intriguing computational prediction, a series of specially designed single and double pulse cavitation bubble imaging experiments as well as the continuous ablation of two metals, gold and silver, are performed and reported in the next section.

### Experiments: Cavitation bubble dynamics and nanoparticle size distributions

There have been few imaging measurements reported in the literature on cavitation bubbles induced by picosecond LAL,^52^ although picosecond pulses come with certain advantages, such as a high nanoparticle productivity.^53^ To support the computational predictions, we designed a series of imaging experiments aimed at searching for possible “signatures” of the large nanoparticles injected into the water environment above the cavitation bubble boundary. The evolution of a cavitation bubble generated by a single 10 ps laser pulse irradiation of a Au target in water and the response of the evolving bubble to a second pulse arriving with a microsecond timescale delay are investigated in time-resolved imaging experiments. The nanoparticle size distributions of the single pulse generated colloids are also analyzed for Au and Ag targets, and the insights into the mechanisms of nanoparticle generation in picosecond LAL are related to the computational predictions.

**Fig. 4:**
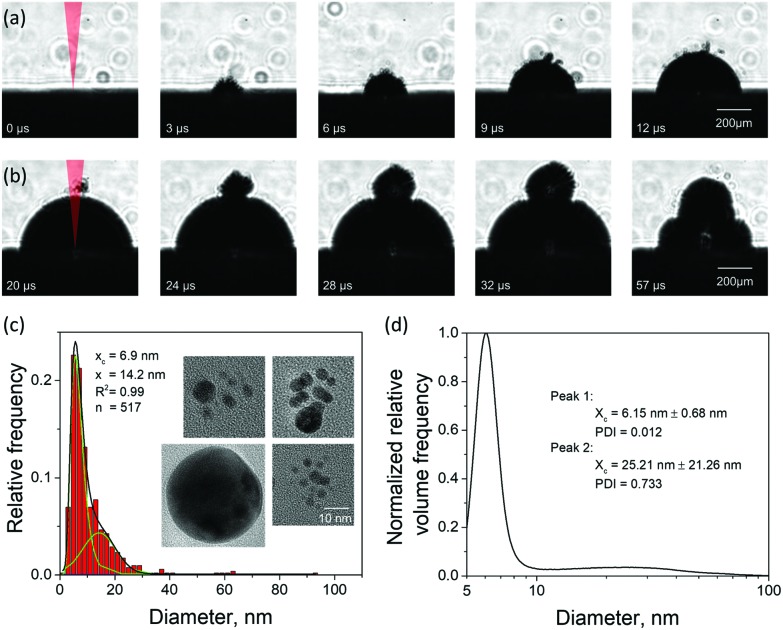
Experimental results on the cavitation bubble dynamics and generation of nanoparticles in LAL of Au targets irradiated by 10 ps laser pulses in water at an incident fluence of 3.4 J cm^–2^ ± 0.51 J cm^–2^ and laser wavelength of 1064 nm. (a) Images of a cavitation bubble with rough boundary (satellite microbubbles) generated by a single laser pulse irradiation taken at regular intervals during the first 12 μs after the laser pulse. (b) Images of the cavitation bubble dynamics modified by a second pulse applied at 20 μs after the first one, *i.e.*, during expansion of the first cavitation bubble. The first four images are separated from each other by 4 μs, and the fifth image is taken 25 μs after the fourth image, *i.e.*, during the shrinking phase. (c) Size distribution of Au nanoparticles generated by single pulse LAL and obtained from analysis of TEM images, with several representative images shown as insets. The scale bar is common for all insets and corresponds to 10 nm. (d) Nanoparticle size distribution obtained through analytical disc centrifugation measurement for nanoparticle solution produced under the same experimental conditions as in (c) but for a continuous ablation with a repetition rate of 200 kHz. Volume frequency is shown to increase the visibility of the second mode.

The emergence of a cavitation bubble following a single pulse irradiation of 10 ps width is shown in Fig. 4a for the first 12 μs after the laser impact. The cavitation bubble exhibits unique features that have not been observed in nanosecond LAL performed in a clean (no ablation products from prior laser pulses) liquid at laser intensities below the threshold for dielectric breakdown^52^ or significant solvent heating,^54^ where the bubble boundaries tend to be sharp and smooth.^20^,^55^ The bubble in Fig. 4a exhibits a rough and diffuse boundary with numerous “microbubbles” protruding out of the boundary of the main bubble. The rough interface, which is already apparent at 3 μs after the laser pulse, gradually evolves into a well-defined hemispherical main bubble surrounded by several satellite microbubbles, some of which can still be seen as late as 20 μs after the pulse (first frame in Fig. 4b).

The appearance of the satellite micro-sized bubbles surrounding the main cavitation bubble may be related to the injection of the large nanoparticles into the dense water region above the precursor of the cavitation bubble observed in the simulations (Fig. 2). Although the simulations can only treat the initial stage of the cavitation bubble expansion, it is reasonable to expect that the large nanoparticles injected into the water environment will stay above the boundary of the bubble during its further expansion. The question on how the existence of large nanoparticles beyond the boundary of the cavitation bubble can result in the formation of microbubbles, however, remains open. As demonstrated in the simulation, the nanoparticles embedded into the dense water environment are expected to cool down below the melting temperature and solidify on the timescale of several nanoseconds. The cooling rate is ensured by the suppression of the formation of an insulating vapor layer around the hot nanoparticles by the high curvature of the nanoparticle-water interface. Therefore, complete thermal equilibration between the nanoparticles and the surrounding water can be expected within a few tens of nanoseconds. Hence, the nanoparticles cannot be expected to serve as sustained heat sources acting to support microscale vapor bubbles on the microsecond timescale. One possible explanation of the satellite microbubbles is that the metal nanoparticles can serve as heterogeneous nucleation sites lowering the nucleation barrier for vapor nucleation in a hot water layer surrounding the expanding cavitation bubble.

Given the ambiguity with association of the satellite microbubbles with metal nanoparticles, the origin of the satellite microbubbles can be further investigated in double pulse stroboscopic videography experiment, illustrated in Fig. 4b. Here, the second picosecond pulse is applied 20 μs after the first one, when the main cavitation bubble is still expanding and satellite microbubbles are visible near the top of the main bubble. The irradiation by the second pulse leads to the appearance of a secondary cavitation bubble that originates at the location of the satellite microbubbles. This observation can be interpreted as evidence in favor of the presence of large nanoparticles beyond the boundaries of the main cavitation bubble. Such nanoparticles could absorb laser light from the second pulse, heat the surrounding water, and result in the emergence of the secondary cavitation bubble.^56^,^57^ As time progresses, the secondary bubble expands, interacts and merges with the main cavitation bubble, thus drastically altering the overall cavitation bubble dynamics.

In order to test alternative explanations of the appearance of the satellite microbubbles, we performed several control experiments, which are summarized in section 5 of the ESI.† Based on these experiments we can exclude self-focusing as a possible origin of the microbubbles. Moreover, we show that these microbubbles do not form if the laser is focused into the liquid instead of the ablation target (Fig. S5a†). It is also evident that a certain threshold fluence needs to be exceeded to observe microbubbles (Fig. S5c†).

While the generation of a secondary cavitation bubble has been observed in double nanosecond laser pulse ablation of silver target in water,^58^ the physical origins of the secondary bubbles generated in the nanosecond and picosecond double-pulse experiments are different. In ref. 58, the secondary cavitation bubble is only observed for interpulse delays that correspond to the early expansion of the first bubble. Contrary to these results, we show that in picosecond ablation a second cavitation bubble is also induced during the collapse phase of the first cavitation bubble (see Fig. S5b in ESI†). The clusters of microbubbles above the main cavitation bubble boundary, responsible for the formation of secondary cavitation bubble reported in the current work, is not observed in ref. 58. Indeed, the atomistic simulations of LAL performed with longer, hundreds of picoseconds to a nanosecond, laser pulses (to be reported elsewhere) show that the nanojetting produced through the cascade of hydrodynamic instabilities discussed in previous section is not activated by the longer pulses, and no large nanoparticles are located beyond the boundary of the primary cavitation bubble.

The combination of the single and double picosecond pulse cavitation bubble imaging results indicates that, in compliance with computational predictions, large (∼10–20 nm) nanoparticles can be implanted into water environment beyond the boundary of the cavitation bubble during picosecond LAL. We note that none of the alternative mechanisms of the nanoparticle generation in short pulse LAL discussed in literature, such as the generation of large nanoparticles through target heating by the laser-induced plasma and/or the mechanical erosion of target surface by the collapsing cavitation bubble,^6^,^7^ can explain the appearance of the nanoparticles in water above the boundary of the cavitation bubble. Large droplets produced through hydrodynamic splashing or radial flow in the molten layer driven by the recoil pressure or temperature gradients generated on the scale of the whole laser spot^59^,^60^ can potentially cross the boundary of the cavitation bubble and inject into the liquid environment. This mechanism of large droplet ejection, however, is clearly undesirable, and the sputtering/splashing regime should be avoided in the nanoparticle generation by LAL.

**Fig. 5:**
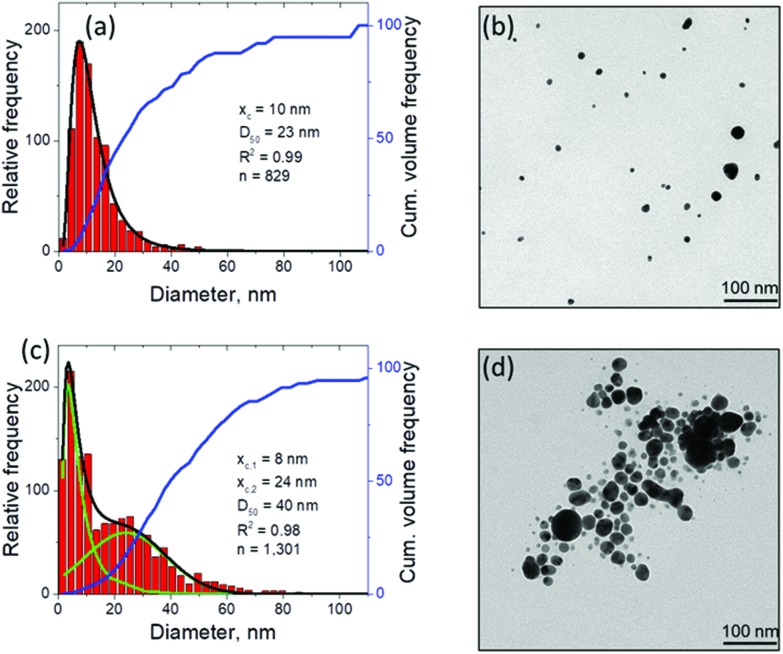
Nanoparticle characterization by means of TEM with image analysis after continuous picosecond LAL synthesis from flat gold (a, b) and silver (c, d) targets in a flow chamber. The laser differs from that used in Fig. 4, for details see Methods section. The black curve in (a) shows the lognormal envelope of the histogram. In (c), the black curve shows the sum of the two underlying fits, a lognormal (*x*_c,1_) and a Gaussian (*x*_c,2_) one, shown in green. Obviously, the predicted bimodality from the computational model is experimentally better reproduced by the ablation of silver compared to the ablation of gold.

An additional connection to the simulation results can be provided through analysis of the nanoparticle size distributions. For the single picosecond laser pulse experiment, the distribution obtained from TEM image analysis is shown, along with several representative images, in Fig. 4c. The envelope of the distribution shows a peak with maximum at ∼4 nm and a tail extending up to ∼40 nm. It is worth noting that for the imaging experiments a gold target was used whereas the simulation is carried out with a silver target. To close this gap, continuous ablation of both silver and gold targets was conducted with a high-power picosecond laser (EdgeWave, see Methods section). In order to minimize the influence of particle re-irradiation, a flow chamber was used to transport the freshly synthesized nanoparticles away from the ablation spot. The number-weighted histograms obtained from TEM image analysis are shown in Fig. 5a and c together with corresponding representative images in Fig. 5b and d. Surprisingly, the bimodality of the gold colloid (Fig. 5a) is much less pronounced compared to the silver colloid (Fig. 5c). In fact, the size distribution of gold particles represents a lognormal envelope (*R*^2^ = 0.99). Nevertheless, this does represent at least two particles size fractions as can be seen from the difference between the center of gravity of the number-weighted lognormal envelope, *x*_c_ = 10 nm, and the mode diameter of the volume-weighted distribution, *D*_50_ = 23 nm. For a monodisperse particle size distribution, these two parameters characterizing the number- and volume-weighted particle size distributions would be equal to each other. Yet, the bimodality of the silver colloid (Fig. 5b) is recognized even more intuitively, as the envelope requires a two-peak fitting (lognormal for small particles and Gaussian for large particles) to obtain an adequate fit (*R*^2^ = 0.98). Overall, the results obtained from the ablation of silver shows a good agreement with the simulation.

The experimental size distributions shown in Fig. 4c and 5 are in a good quantitative agreement with the results of the simulations, where the volume-weighted distribution of small nanoparticles generated through the nucleation and growth in the metal-water mixing region peaks around 4 nm (Supplementary Fig. S4†) and the large nanoparticles produced through the nanojet disintegration have diameters of 12 nm, 15 nm, and 19 nm. The size of the large nanoparticles in the simulation may be affected by the relatively small lateral size of the computational system, which only allows for the emission of a single nanojet. Under experimental conditions, the particles ejected from neighboring nanojets may coalesce, while the nanoparticles that end up in the low-density region (*e.g.*, nanoparticle #3 in Fig. 3a) may grow with time by consuming the surrounding Ag atoms and clusters.

## Conclusions

Atomistic modeling of picosecond laser ablation of Ag in water combined with cavitation bubble imaging experiments performed for Au targets and continuous ablation for Au and Ag targets provide new insights into the mechanisms of nanoparticle formation in picosecond LAL and reveal a complex sequence of processes responsible for generation of two distinct size groups of nanoparticles, thus explaining the origin of the commonly observed bimodal nanoparticle size distribution.

The results of a large-scale atomistic simulation, performed at a laser fluence three times above the phase explosion threshold in vacuum, provide convincing evidence of the critical role the formation of a transient hot molten metal layer at the interface with water environment plays in the nanoparticle generation. The water in contact with the hot metal layer is brought to the supercritical state and expands into a low-density metal-water mixing region that serves as a precursor for the formation of a cavitation bubble. The thermodynamic conditions in the low-density mixing region are amenable to rapid nucleation and growth of small (below 10 nm) nanoparticles from Ag atoms that are continuously evaporating from the hot molten metal layer. In addition to serving as a source of Ag atoms for condensation of small nanoparticles in the mixing region, the hot molten layer itself has limited stability and can readily disintegrate into larger (10–20 nm) nanoparticles through series of hydrodynamic instabilities. In particular, rapid deceleration of the molten metal layer by pressure exerted by supercritical water leads to Rayleigh-Taylor instability of the interface and produces extensive nanoscale interfacial roughness on a timescale of hundreds of picoseconds. The impact from new metal droplets or spalled layers joining the hot molten metal layer at a later time can further destabilize the interface by inducing Richtmyer-Meshkov instability of the roughened interface. The latter can lead to the formation of nanojets launching large metal droplets past the low-density mixing region directly into dense and relatively cold water environment.

The direct injection of large nanoparticles into liquid beyond the cavitation bubble boundary predicted in the simulation is directly confirmed in the cavitation bubble imaging experiments, where small satellite microbubbles surrounding the main cavitation bubble are observed upon single laser pulse irradiation of a Au target. The formation of secondary bubbles originating from the satellite microbubbles upon properly timed second picosecond laser pulse irradiation further confirms the association of the microbubbles with large nanoparticles. The nanoparticle size distributions obtained through the analysis of TEM images and analytical disc centrifugation for Au and Ag targets show the presence of both small (less than 10 nm) and large (tens of nm) nanoparticles, and are consistent with the distributions predicted in the simulation. The good quantitative agreement between the simulation and experiment supports the association of the two groups of nanoparticles with two distinct mechanisms of the nanoparticle formation in picosecond LAL, *i.e.*, the nucleation and growth of small nanoparticles in the metal-water mixing region and generation of larger nanoparticles through the breakup of the superheated molten metal layer generated at the plume-water interface.

## Methods

### Computational method

The simulation reported in this paper is performed for a Ag bulk target covered by water and irradiated by a 10 ps laser pulse at an absorbed laser fluence of 600 mJ cm^–2^. A hybrid model^35^,^37^,^40^,^47^ combining continuum level description of laser excitation of conduction band electrons and subsequent electron-phonon equilibration based on Two-Temperature Model (TTM)^61^ with fully atomistic description of laser-induced structural and phase transformations in metal targets is used in the calculations. The model has been successfully applied in simulations of short pulse laser interactions with metals in vacuum, *e.g.*, ref. 35, 37, 47 and 62. The direct application of the conventional all-atom MD representation of water in large-scale simulations of LAL, however, is not feasible due to the high computational cost. Thus, a coarse-grained MD representation of a part of the liquid environment adjacent to the metal target is adapted in this work, whereas the mechanical confinement provided by the bulk of a thick water overlayer is represented through a dynamic boundary condition applied at the outer boundary of the coarse-grained MD region. The coarse-grained MD model combines the breathing sphere model developed for simulations of laser interaction with molecular systems^63^,^64^ with a heat bath approach that associates an internal energy variable with each coarse-grained particle.^29^–^31^,^65^,^66^

A schematic representation of the computational system used in the simulation is shown in Supplementary Fig. S1.† The computational system represents a small region within the laser spot, and periodic boundary conditions are applied in the lateral directions, parallel to the surface of the target. The dimensions of the computational system in these directions are 49.4 nm × 49.4 nm. The depth of the surface part of the Ag target represented with atomistic resolution is 500 nm, which corresponds to 70 million atoms interacting *via* EAM Ag potential.^67^ The heat transfer in the deeper part of the target is described by the TTM equations solved for lattice and electron temperatures down to the depth of 6 μm. The part of the water overlayer represented by the coarse-grained MD is 300 nm thick and consists of 8.5 million coarse-grained particles. To match the experimental conditions, the dynamic acoustic impedance matching boundary conditions imposed at the top and bottom of the computational domain are designed to mimic non-reflecting propagation of the laser-induced pressure waves through the boundaries of the computational domain.^32^,^68^ These boundary conditions implicitly simulate a sufficiently thick liquid overlayer and metal target, so that reflections of the laser-induced pressure waves from the free surface of a thick liquid overlayer and the opposite side of the metal target do not play any significant role in the generation of nanoparticles. The parameters of the hybrid atomistic – coarse-grained MD model tailored for simulation of short pulse laser interaction with Ag in water environment and additional details of the computational setup are provided in ESI.†

### Experimental method

A Fuego laser system from Time-Bandwidth with a pulse duration of 10 ps, a pulse energy of 120 μJ, and a wavelength of 1064 nm was used in the single and double pulse experiments. The camera system for imaging of the cavitation bubbles was Phantom v1210 from Vision Research Inc. The image refreshing rate was 240 300 fps at a resolution of 128 × 128 pixels.

The single pulse experiments are performed for Au rather than Ag targets used in the simulations. While the nanoparticle formation can be expected to be similar for both of the noble metals, the use of the more inert Au eliminates any contribution of oxidation during the ablation process, which is also not included in the simulation of Ag ablation. The deionized water used for the single pulse experiments has a conductivity of 0.055 μS cm^–1^ and was adjusted to pH 8 to stabilize the nanoparticles with a sodium phosphate buffer (0.1 mM).

In both single-pulse and double-pulse experiments, N-BK7 glass cubes (top open) with an outer dimension of 11 × 11 × 11 mm and a wall thickness of 2 mm were used. The gold target was attached to an inner sidewall of a glass cube. The liquid volume was 300 μl, while the liquid thickness between the gold target and the glass was 6 mm. After each pulse (or double-pulse), the chamber was moved 200 μm orthogonally to the laser beam and after several cleaning steps of the chamber, the liquid volume was changed. The laser beam was focused horizontally on the target by means of a focusing lens with a 100 mm focal length. The resulting focus had a diameter of 67 μm ± 5 μm and was determined by microscopy applying the zero-damage method.^69^ This leads to a fluence of 3.4 J cm^–2^ ± 0.51 J cm^–2^. The incident fluence applied in experiment, when converted to the absorbed fluence through a TTM simulation performed with electron temperature dependent electronic heat capacity, electron-phonon coupling,^70^ thermal conductivity^71^ and reflectivity^72^ is estimated to be between 1000 and 2000 mJ cm^–2^ (see ESI† for details of the calculations), *i.e.*, comparable to the fluence of 600 mJ cm^–2^ used in the simulation of LAL of Ag discussed in this paper.

For continuous ablation of gold and silver, a laser system from EdgeWave was used. It has a pulse width of 12 ps, a pulse energy of 1.3 mJ, and a wavelength of 1064 nm. The repetition rate was set to 1 kHz, while the ablation process was carried out in a flow chamber made of Teflon. A scanning system was used to move the laser beam on the target surface to avoid fast penetration of the target and to bypass the previous cavitation bubbles. The spot size on silver was measured to be 350 μm in diameter and reproduced the Gaussian shape of the laser beam. The volume flow of water was set to 25 ml min^–1^ to minimize re-irradiation of freshly synthesized nanoparticles. MilliQ water (18.2 MΩ) was used for the continuous synthesis of gold and silver colloids.

TEM images were obtained with a JEM-2200FS from JOEL USA, Inc. Prior to drop-casting onto the Cu grids, the samples from continuous ablation were mixed 1 : 1 with an aqueous solution of PVP (0.25 g l^–1^, 58 000 g mol^–1^) to minimize aggregation of nanoparticles upon evaporation of the liquid.

## Conflicts of interest

The authors declare no conflict of interest.

## Supplementary Material

Supplementary information
