# Supplementary material for: Two mechanisms of nanoparticle generation in picosecond laser ablation in liquids: the origin of the bimodal size distribution
Source: Nanoscale. 2018 Mar 8;10(15):6900–10. doi: 10.1039/c7nr08614h (PMC6637654; doi:10.1039/c7nr08614h)
Supplement: Supplementary file 1 [file NR-010-C7NR08614H-s001.pdf]

Electronic Supplementary Material (ESI) for Nanoscale.
This journal is © The Royal Society of Chemistry 2018

SUPPLEMENTARY INFORMATION FOR

Two mechanisms of nanoparticle generation in picosecond laser ablation in liquids: Connection to bimodal size distribution

Cheng-Yu Shih,[1] René Streubel,[2] Johannes Heberle,[3] Alexander Letzel,[2] Maxim V. Shugaev,[1] Chengping Wu,[1] Michael Schmidt,[3] Bilal Gökce,[2] Stephan Barcikowski,[2] and Leonid V. Zhigilei[1]

[1] Department of Materials Science and Engineering, University of Virginia, 395 McCormick Road, Charlottesville, Virginia 22904-4745, USA

[2] Technical Chemistry I and Center for Nanointegration Duisburg-Essen (CENIDE), University of Duisburg-Essen, Universitaetsstr. 7, Essen 45141, Germany

[3] Institute of Photonic Technologies, Friedrich-Alexander University Erlangen-Nürnberg, Konrad-Zuse-Straße 3/5, Erlangen 91052, Germany

Contents

1. Details of computational setup and Supplementary Figure S1
2. Estimation of the experimental absorbed laser fluence and Supplementary Figure S2
3. Supplementary Figure S3: Rayleigh-Taylor instability of the plume-water interface
4. Formation of small clusters via vapor condensation and growth, Supplementary Figure S4
5. Control double pulse experiments aiming to clarify the nature of the satellite bubbles and their interaction with the second pulse, Supplementary Figure S5

1. Details of computational setup and Supplementary Figure S1

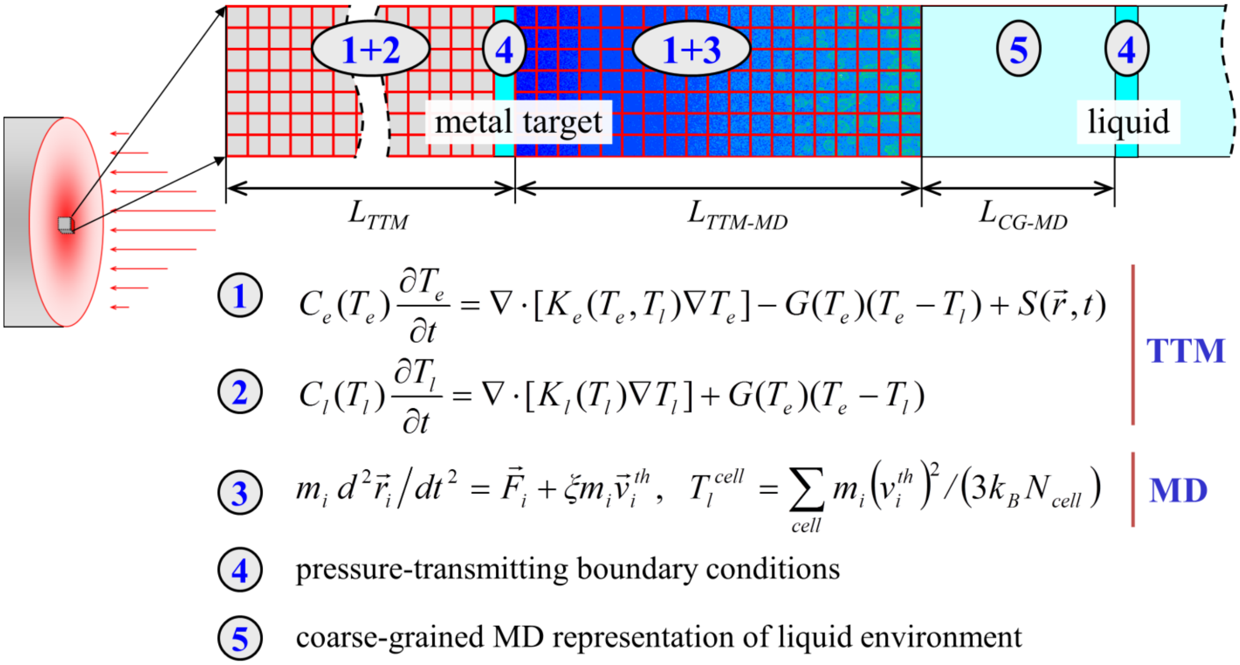

Figure S1. Schematic representation of the combined continuum - coarse-grained - atomistic model for simulation of laser interactions with metals in liquid environment. The top part of the metal target is represented by TTM-MD model, the temperature evolution in the deeper part of the target is described by TTM equations, and part of the liquid environment adjacent to the metal surface is simulated with a coarse-grained MD model. At the bottom of the TTM-MD and on the top of the coarse-grained MD regions the pressure wave transmitting boundary conditions are imposed. Both the Ag target and the liquid overlayer are assumed to be sufficiently thick to ensure that any effects caused by the reflection of the laser-induced pressure waves from the outer surfaces of the target and overlayer can be neglected. The computational system represents a small region within the laser spot and periodic boundary conditions are applied in the lateral directions, parallel to the surface of the target. The spatial discretization in the continuum part of the model and the dimensions of the atomistic and continuum regions are not drawn to scale.

The simulations reported in this paper are performed with a hybrid computational model combining a coarse-grained representation of liquid, a fully atomistic description of laser interaction with metal targets, and acoustic impedance matching boundary conditions, designed to mimic the non-reflecting propagation of the laser-induced pressure waves through the boundaries of the computational domain. A schematic representation of the computational system is shown in Figure S1. The computational setup is designed and parametrized for a bulk Ag target covered by water and irradiated by a picosecond laser pulse. A brief description of the main components of the computational model as well as details of the computational setup are provided below.

TTM-MD model for laser interactions with metals:

Although the MD method is capable of providing detailed information on the microscopic mechanisms of laser ablation, several modifications have to be made in order to apply the classical MD for simulations of laser interactions with metals. In particular, a realistic description of the laser coupling to the target material, the kinetics of thermalization of the absorbed laser energy, and the fast electron heat conduction should be incorporated into the MD technique. These processes can be accounted for by incorporating the MD method into the general framework of the two-temperature model (TTM)[1, 2] commonly used in the simulations of short pulse lase interactions with metals. The idea of the combined TTM-MD model[3-5] is schematically illustrated in Figure S1 and is briefly explained below.

In the original TTM, the time evolution of the lattice and electron temperatures, T_l and T_e, is described by two coupled differential equations (Eqs. (1) and (2) in Figure S1) that account for the electron heat conduction in the metal target and the energy exchange between the electrons and atomic vibrations. In the combined TTM-MD method, MD substitutes the TTM equation for the lattice temperature in the surface region of the target, where laser-induced structural and phase transformations take place. The diffusion equation for the electron temperature, T_e, is solved by a finite difference method simultaneously with MD integration of the equations of motion of atoms. The cells in the finite difference discretization are related to the corresponding volumes of the MD system, and the local lattice temperature, T_l^{cell}, is defined for each cell from the average kinetic energy of thermal motion of atoms. The electron temperature enters a coupling term, $\xi m_i \vec{v}_i^{\,th}$, that is added to the MD equations of motion to account for the energy exchange between the electrons and atomic vibrations. In this coupling term, ξ is a coefficient that depends on the instantaneous

difference between the local lattice and electron temperatures as well as the strength of the electron-phonon coupling,[3] $\vec{v}_i^{th}$ is the thermal velocity of the atom defined as $\vec{v}_i^{th} = \vec{v}_i - \vec{v}^c$, where $\vec{v}_i$ is the actual velocity of atom *i* and $\vec{v}^c$ is the velocity of the center of mass of a cell to which the atom *i* belongs. The expansion, density variation and, at higher fluences, disintegration of the irradiated target predicted in the MD part of the model are accounted for through the corresponding changes of the parameters of the TTM equation for electron temperature. The three-dimensional solution of the diffusion equation for T_e is used in simulations of laser spallation and ablation,[5-7] where the dynamic material decomposition may result in lateral density and temperature variations.

In the continuum part of the model, beyond the surface region represented by the MD method, the electron heat conduction and the energy exchange between the electrons and the lattice are described by the conventional TTM equations. A dynamic pressure-transmitting boundary condition[8, 9] is applied at the bottom of the MD part of the system (marked as ④ in Figure S1) to ensure non-reflecting propagation of the laser-induced stress wave from the MD region of the computational system to the bulk of the target. The energy carried away by the stress wave is monitored, allowing for control over the total energy conservation in the combined model.[10]

The electron temperature dependences of the thermophysical material properties included in the TTM equation for the electron temperature (electron-phonon coupling factor *G* and electron heat capacity C_e, see Figure S1) are taken in the forms that account for the thermal excitation from the electron states below the Fermi level.[11] The electron thermal conductivity is described by the Drude model relationship, $K_e(T_e,T_l) = v^2 C_e(T_e)\tau_e(T_e,T_l)/3$, where $C_e(T_e)$ is the electron heat capacity, v^2 is the mean square velocity of the electrons contributing to the electron heat conductivity, approximated in this work as the Fermi velocity squared, v_F^2, and $\tau_e(T_e,T_l)$ is the total electron scattering time defined by the electron-electron and electron-phonon scattering rates, $1/\tau_e = 1/\tau_{e-e} + 1/\tau_{e-ph} = AT_e^2 + BT_l$. The value of the coefficient *A*, 3.57×10^6 $s^{-1}K^{-2}$, is estimated[12] within the free electron model, following the approach suggested in Ref. 13. The value of the coefficient *B*, 1.12×10^{11} $s^{-1}K^{-1}$ is obtained from the experimental value of the thermal conductivity of solid Ag at the melting temperature, 363 $Wm^{-1}K^{-1}$.[14]

Material properties predicted by EAM Ag potential:

The interatomic interactions in the MD part of the model are described by the embedded atom method (EAM) potential with the functional form and parameterization developed in Ref. 15. A cut-off function suggested in Ref. 16 is added to the potential to smoothly bring the interaction energies and forces to zero at interatomic distance of 5.5 Å. Although the potential is fitted to low-temperature values of the equilibrium lattice constant, sublimation energy, elastic constants, and vacancy formation energy, it also provides a good description of high-temperature thermodynamic properties of Ag[17] relevant to the simulation of laser-induced processes. In particular, the equilibrium melting temperature, T_m, determined in liquid–crystal coexistence simulations, is 1139 ± 2 K,[12] about 8% below the experimental values of 1235 K.[18] The threshold temperature for the onset of the explosive phase separation into liquid and vapor, T^*, determined in simulations of slow heating of a metastable liquid, is found to be ~3450 K at zero pressure and ~4850 K at 0.5 GPa.[19] The onset of the phase explosion can be expected at 10% below the critical temperature[20-22] and the values of T^* calculated for the EAM Ag material are not in conflict with the range of experimental values of the critical temperature of Ag spanning from 4300 K to 7500 K.[23]

Coarse-grained representation of liquid environment:

The direct application of the conventional all-atom MD representation of liquids in large-scale simulations of laser processing or ablation is not feasible due to the high computational cost. Thus, a coarse-grained representation of the liquid environment,[24, 25] where each particle represents several molecules, is adapted in this work. The coarse-graining reduces the number of degrees of freedom that have to be treated in the MD simulations and significantly increases the time and length scales accessible for the simulations. At the same time, however, the smaller number of the dynamic degrees of freedom results in a severe underestimation of the heat capacity of the liquid. To resolve this problem, the degrees of freedom that are missing in the coarse-grained model are accounted for through a heat bath approach that associates an internal energy variable with each coarse-grained particle.[26-28] The energy exchange between the internal (implicit) and dynamic (explicit) degrees of freedom are controlled by the dynamic coupling between the translational degrees of freedom and the vibrational (breathing) mode associated with each coarse-grained particle (the particles are allowed to change their radii, or to "breath" [25, 28]). The energy exchange is implemented through a damping or viscosity force applied to the breathing mode, which

connects it to the energy bath with capacity chosen to reproduce the real heat capacity of the group of atoms represented by each coarse-grained particle. Effectively, the breathing mode serves as a "gate" for accessing the energy stored in the molecular heat bath.

The first implementation of the coarse-grained model with heat bath approach was recently developed for water and applied to simulations of laser interactions with water-lysozyme system.[28] Each coarse-grained particle in the model has a mass of 50 Da and represents about three real water molecules. The potential describing the inter-particle interactions is provided in Ref. 28 and the parameters of the potential are selected to ensure a satisfactory semi-quantitative description of experimental properties of water. While one cannot expect the coarse-grained model to provide an accurate representation of all the structural and thermodynamic properties of water, the key physical properties predicted by the model, such as density, speed of sound, bulk modulus, viscosity, surface energy, melting temperature, critical temperature, and critical density, are found to not deviate from the experimental values by more than 25%.[29]

The interactions between Ag atoms and the coarse-grained water particles are described by the Lennard-Jones (LJ) potential with the following length and energy parameters: σ = 1.92 Å and ε = 0.145 eV. The parameters are fitted to match the diffusion of metal atoms and small clusters in water predicted by the Stoke-Einstein equation at 300 K and calculated in constant-temperature and constant-volume MD simulations. Furthermore, the values of σ and ε are chosen to ensure that the values of the equilibrium O-Ag distance and the adsorption energy of water on a Ag surface predicted in *ab-initio* simulations[30-33] are roughly reproduced in the coarse-grained model. Note that, while it is possible to incorporate the description of chemical reactions into the framework of coarse-grained MD model,[24, 34, 35] we have not included descriptions of oxidation or other chemical reactions[36] in the version of the model used in the present study.

2. Estimation of the experimental absorbed laser fluence

To facilitate comparison of the experimental observations with computational predictions, the incident laser fluence applied in the experimental part of this study is converted to the absorbed laser fluence with the help of TTM simulation accounting for the electron temperature dependence of the reflectivity and optical absorption coefficient, as well as for the kinetics of surface melting as described in Ref. 37.

In the experiment, Au target is irradiated by a 10 ps laser pulse at the wavelength of λ = 1064 nm and incident fluence of 3400 mJ/cm^2. The irradiation of the target by a laser pulse is represented through a source term added to the TTM equation for the electron temperature. The source term accounts for the excitation of the conduction band electrons by a laser pulse with a Gaussian temporal profile and reproduces attenuation of laser intensity with depth under the surface according to Beer–Lambert law.

The electron temperature dependences of the electron-phonon coupling factor and electron heat capacity are taken in the forms that account for the thermal excitation from the electron states below the Fermi level.[11] The electron thermal conductivity of Au is approximated as suggested in Ref. 38. The lattice heat capacity of Au is taken to be 25.41 Jmol^{-1}K^{-1}, density is 19300 kg/m^3, melting temperature is 1337 K, and the heat of fusion is 12.7 kJ/mol.[18]

The reflectivity and the absorption depth are calculated based on complex permittivity coefficient according to:

$$R = \left| \frac{\sqrt{\varepsilon_{Au}} - \sqrt{\varepsilon_w}}{\sqrt{\varepsilon_{Au}} + \sqrt{\varepsilon_w}} \right|^2, \; l_{abs} = \frac{\lambda}{4\pi \operatorname{Im}\left(\sqrt{\varepsilon_{Au}}\right)},$$

where ε_{Au} and ε_w are relative permittivities of Au and water, ε_w = 1.78.[18] The complex permittivity of Au is parametrized based on Ref. 39:

$$\varepsilon_{Au} = \varepsilon_\infty - \frac{\omega_p^2}{\omega \cdot \left(\omega + i\,\nu_{ef}\right)} - \frac{f\Omega_L^2}{\left(\omega^2 - \Omega_L^2\right) + i\Gamma_L \omega},$$

where Γ_L = 0.659×10^{15} Hz and Ω_L = 4.085×10^{15} Hz are the oscillator strength and the spectral width of the Lorentz oscillators, respectively, f = 1.09 is the weighting factor, ω_p = 1.328×10^{16} Hz

is the plasma frequency, ε_∞ = 5.9673 is a dc dielectric function, $\omega = 2\pi c / \lambda$ is the angular laser frequency, ν_{ef} is an effective electron collision frequency, $\nu_{ef} = \min(\nu_e, \nu_c)$. The electronic collision frequency is the sum of the electron-electron and electron-phonon collision frequencies $\nu_e = AT_e^2 + BT_l$, A = 1.2×10^7 K^{-2}s^{-1} and B = 1.23×10^{11} K^{-1}s^{-1}.[40] The maximum critical electron collision frequency is estimated as $\nu_c = (4\pi n_0 / 3)^{1/3} \sqrt{v_f^2 + k_B T_e / m_e}$ [41], n_0 = 5.90×10^{28} m^{-3}, v_f = 1.40×10^6 m/s.

To account for the energy transport occurring before the thermalization of the excited electrons, the optical absorption depth, l_{abs}, is combined with the effective depth of the "ballistic" energy transport, l_b, yielding the effective range of the laser energy deposition and redistribution by the non-thermal electrons, $l_{eff} = l_{abs} + l_b$. The ballistic range is roughly estimated as a product of the Fermi velocity and the Drude relaxation time, $l_b = v_f / \nu_e$. In the TTM simulation, the instantaneous value of reflectivity is defined by the evolving electron and lattice temperatures on the surface, while the energy deposition fully accounts for the variation of l_{abs} and l_b with depth under the surface. As an example of the strong dependence of the reflectivity and the effective range of the laser energy deposition on the electron temperature, the magnitudes of R and l_{eff} are shown in Figure S2a for a broad range of electron temperatures and the lattice temperature fixed at 300 K.

The evolution of the surface temperature obtained in a TTM simulation is shown in Figure S2b. At an incident laser fluence 3400 mJ/cm^2, the absorbed fluence predicted in the simulation is 1940 mJ/cm^2. Since reflectivity of Au rapidly drops with increase of the electron temperature, the absorbed laser fluence and, therefore, the increase of the electron and lattice surface temperatures is much larger than that predicted in a simulation performed with constant optical properties of Au, shown as dashed lines in Figure S2b. In the latter case, the reflectivity of water-gold interface is assumed to be 0.972, the optical absorption depth is 12 nm,[42] and the temperature dependent ballistic range, similar to the simulation discussed above, is used. Due to the constant high value of reflectivity, the absorbed laser fluence is found to be 93.8 mJ/cm^2. On the other hand, if the ballistic range is fixed at 100 nm,[43] and the variation of the optical properties are taken into account as discussed above, the predicted absorbed fluence becomes ~1030 mJ/cm^2.

The high sensitivity of the predicted value of the absorbed fluence to the temperature dependence of the ballistic range suggests that the value of 1940 mJ/cm^2 can be considered to be an upper bound estimate of the real value. Indeed, the assumption of the electron equilibration within the characteristic time of a single scattering event is likely to result in a substantial underestimation of the ballistic range. The longer ballistic range leads to a slower heating of the surface and, as a result, prolongs the time when the value of reflectivity remains at a high level. Given the uncertainty in the range of the ballistic energy redistribution, we conclude that the absorbed laser fluence under experimental conditions is within the range of 1000 to 2000 mJ/cm^2.

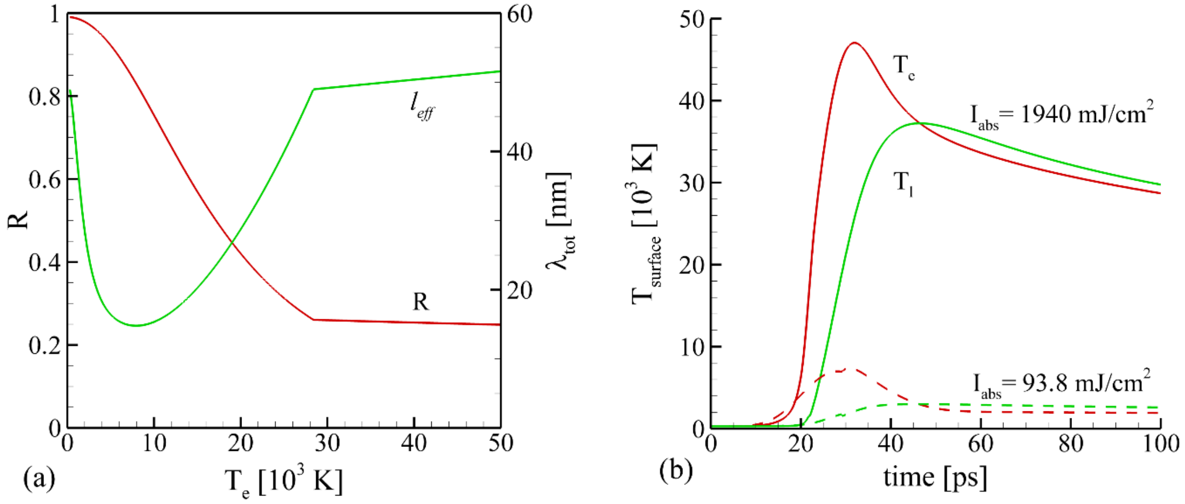

Figure S2. The dependence of the reflectivity and the total absorption depth on the electron temperature at a fixed lattice temperature of 300 K (a) and the evolution of electron and lattice temperatures on the surface of Au target irradiated by a 10 ps laser pulse at an incident fluence of 3400 mJ/cm^2 (b). In (a), red and green lines show the reflectivity and the total effective range of the laser energy deposition, respectively. In (b), red and green lines depict the electron and lattice temperatures, respectively. The solid lines in (b) correspond to the results obtained with a model accounting for the temperature dependence of the ballistic range and optical properties of Au target. The dashed lines in (b) correspond to results of a simulation performed for constant optical properties.

3. Supplementary Figure S3: Rayleigh-Taylor instability of the plume-water interface

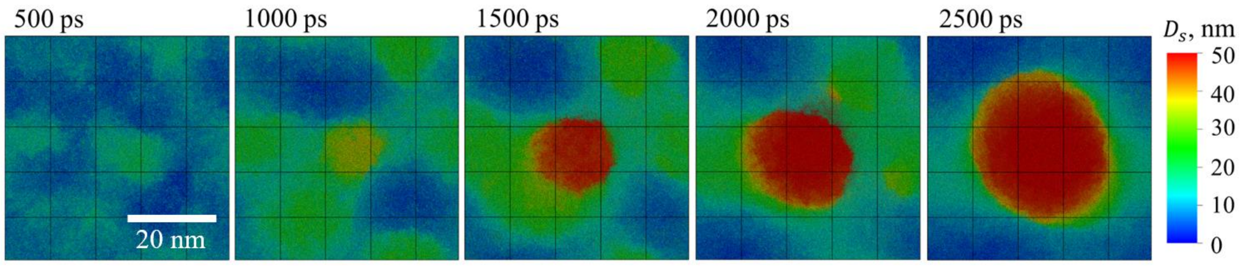

Figure S3. Topographic images of the interface between the hot metal layer and water shown for different times in the simulation illustrated by snapshots in Figure 2. The color shows the relative height of the topographical features from the surface.

4. Formation of small clusters via vapor condensation and growth, Supplementary Figure S4

The analysis of the evolution of sizes of the metal clusters and nanoparticles in the Ag-water mixing region is performed with a cluster identification algorithm applied to atomic configurations generated in the simulation between 100 ps and 5500 ps after the laser pulse, with a 100 ps interval. The three large nanoparticles separated from the liquid jet are not considered in this analysis. The evolution of the cumulative number of Ag atoms present above the liquid layer as individual atoms (vapor) and small atomic clusters with the diameter below 1 nm (less than 30 atoms) as well as the larger clusters that we denote as nanoparticles is shown in Figure S4a. While the total number of Ag atoms in the mixing region steadily increases due to the continuous evaporation from the hot molten metal layer, the number of atoms in the Ag vapor and atomic clusters stays at an approximately the same level starting from 3 ns, and the increase in the total number of Ag atoms in the mixing region is largely sustained by the growing populations of nanometer-scale particles. Overall, the nanoparticle size distribution broadens and shifts to the larger sizes as time progresses, as shown in Figure S4c,d. As can be seen from the snapshots of the metal-water mixing region shown in Figure 2, the largest nanoparticles formed through the nucleation and growth are mostly found in the middle part of the mixing region, where the sufficiently low temperature of the water environment and the high Ag vapor concentration provide the optimum conditions for condensation into Ag nanoparticles.

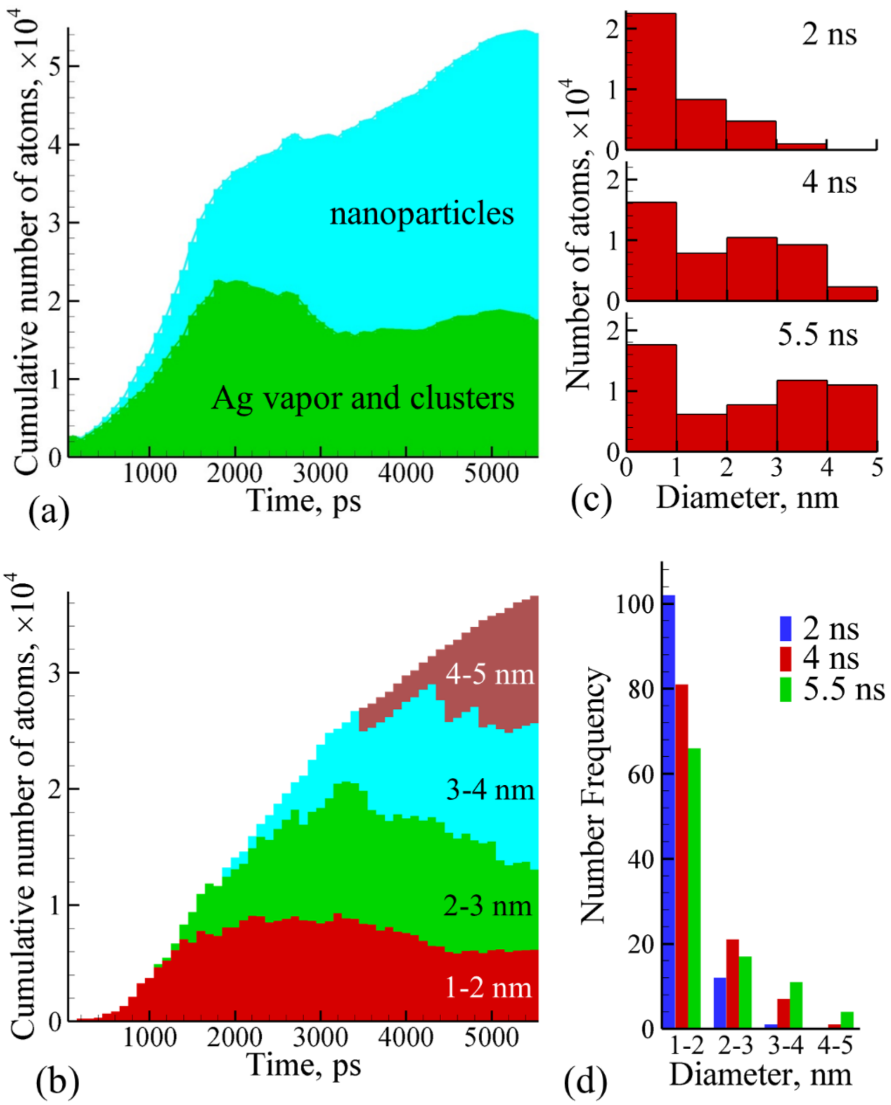

Figure S4. The results of the cluster analysis of the ablation plume generated in the simulation of laser ablation of a bulk Ag target irradiated in water by a 10 ps laser pulse at an absorbed fluence of 600 mJ/cm^2. The cumulative number of individual Ag atoms and small clusters with diameters less than 1 nm (green) and atoms that belong to Ag nanoparticles with diameters above 1 nm (blue) are shown in (a). The cumulative numbers of atoms that belong to nanoparticles of different sizes (above 1 nm) are shown in (b). The number of atoms in nanoparticles of different sizes (above 1 nm) are also shown as histograms for 2, 4 and 5.5 ns in (c). The number of nanoparticles of different sizes is also shown for the same moments of time in (d).

5. Control double pulse experiments aiming to clarify the nature of the satellite bubbles and their interaction with the second pulse, Supplementary Figure S5

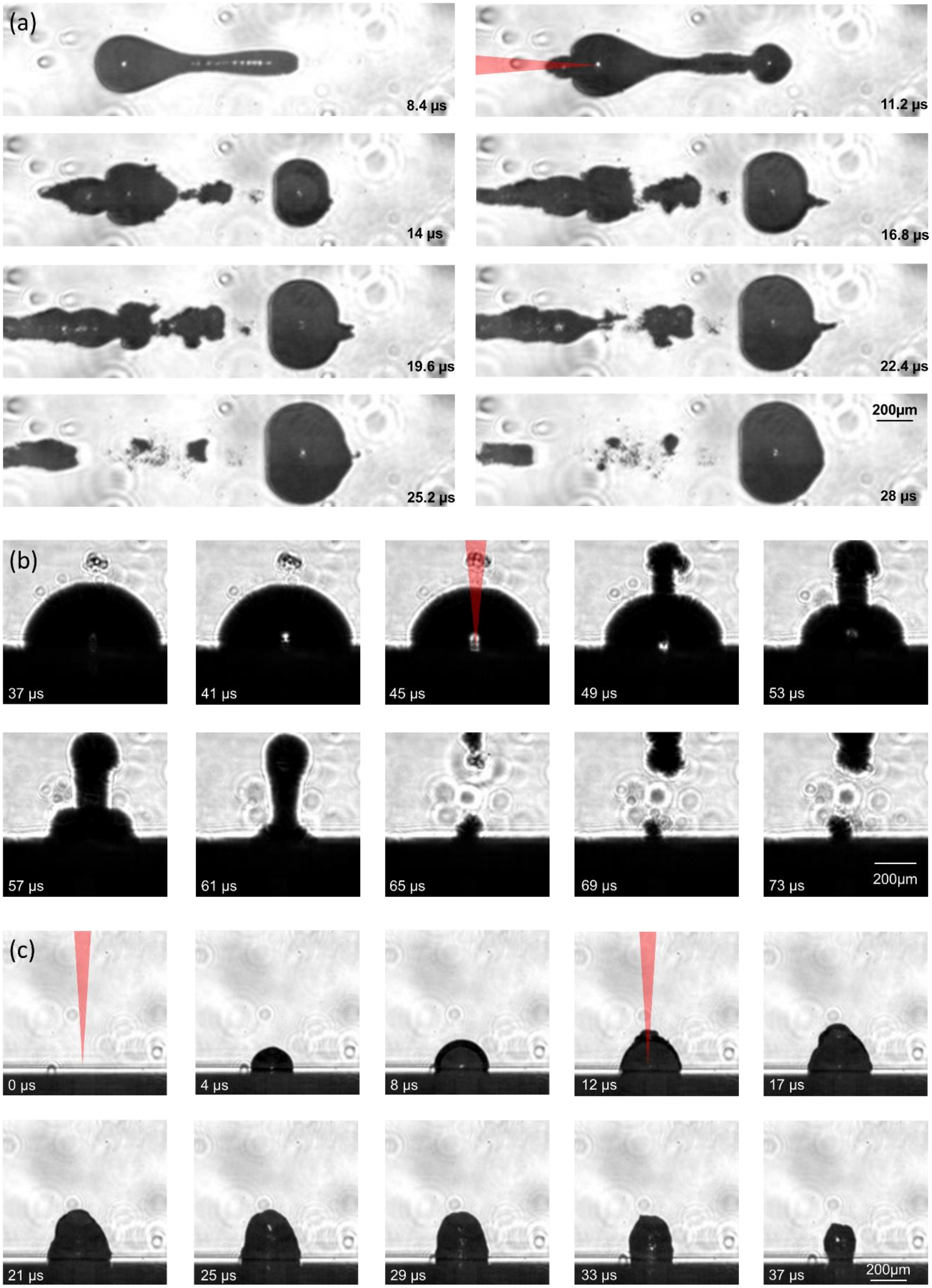

Figure S5. Images of cavitation bubble dynamics in three double pulse control experiments performed to clarify the impact of the satellite bubbles on the second pulse interaction with the original cavitation bubble. The experimental conditions are as described in Figure 4.

(a) Double pulse ablation in a 0.1 mM sodium phosphate buffer solution with no metal target present. No satellite micro bubbles in front of the cavitation bubble (picture one) are formed. Due to the absence of these microbubbles, the second laser pulse (10 µs after the first pulse) reaches the cavitation bubble`s outer boundary with full power and the change in the refractive index leads to a slight shift of the self-focusing plane. This results in the evolution of a second cavitation bubble between the boundary of the first cavitation bubble and the liquid. Thereby, we can visualize minor changes in the beam path due to the shifting of the self-focusing plane.

(b) Double pulse ablation of a Au target under the same irradiation conditions as in Fig. 4, but for longer delay of the second pulse applied 45 µs after the first pulse, *i.e.*, during the collapse phase of the first cavitation bubble. The first image shows the maximum expansion of the cavitation bubble. Shortly after the third image, the second laser pulse hits the cavitation bubble. Image four shows the evolution of a second cavitation bubble due to the irradiation of the satellite microbubbles above the first cavitation bubble. The following images show the subsequent cavitation bubble dynamics. The occurrence of the cylindrical shaped bubble between the expanding second cavitation bubble and the collapsing first cavitation bubble is probably a self-focusing effect comparable to that in Figure S5a, image one. Accordingly, it is clear that the satellite microbubbles are generated only in the presence of a metal target (compare Figure 4b and S5b with S5a).

(c) Double pulse ablation of a Au target. The first pulse has a fluence of 1.2 J/cm^2, the second pulse, which is delayed by 10 µs, has a fluence of 4 J/cm^2. We speculate that due to the low fluence of the first pulse, less particles are produced and, hence, no satellite bubbles formed. Comparable to the situation in Figure S5a, the energy of the second pulse is deposited into the cavitation bubble leading to the expansion of a second cavitation bubble. However, unlike Figure S5a where the residual laser energy can propagate freely into the interior of the first cavitation bubble, the residual laser energy is attenuated by the nanoparticles present inside the cavitation bubble. Finally, the remaining laser energy reaches the target, which leads to a second ablation. This produces an ablation plume expanding in the gas phase environment of the interior of the first cavitation bubble

and, unlike in Figure 4b and S5b, the resulting nanoparticles cannot penetrate the extended phase boundary of the first cavitation bubble, and no satellite microbubbles occur.

References

1. M. I. Kaganov, I. M. Lifshitz and L. V. Tanatarov, *Sov. Phys. JETP* 1957, **4**, 173-178.
2. S. I. Anisimov, B. L. Kapeliovich and T. L. Perel'man, *Sov. Phys. JETP*, 1974, **39**, 375-377.
3. D. S. Ivanov and L. V. Zhigilei, *Phys. Rev. B*, 2003, **68**, 064114.
4. D. A. Thomas, Z. Lin, L. V. Zhigilei, E. L. Gurevich, S. Kittel and R. Hergenröder, *Appl. Surf. Sci.*, 2009, **255**, 9605-9612.
5. C. Wu and L. V. Zhigilei, *Appl. Phys. A*, 2014, **114**, 11-32.
6. S. Noël, J. Hermann and T. Itina, *Appl. Surf. Sci.*, 2007, **253**, 6310-6315.
7. T. E. Itina, K. Gouriet, L. V. Zhigilei, S. Noël, J. Hermann and M. Sentis, *Appl. Surf. Sci.*, 2007, **253**, 7656-7661.
8. C. Schafer, H. M. Urbassek, L. V. Zhigilei and B. J. Garrison, *Comput. Mater. Sci,*, 2002, **24**, 421-429.
9. L. V. Zhigilei and B. J. Garrison, *Mat. Res. Soc. Symp. Proc.*, 1999, **538**, 491-496.
10. L. V. Zhigilei and D. S. Ivanov, *Appl. Surf. Sci.*, 2005, **248**, 433-439.
11. Z. Lin, L. V. Zhigilei and V. Celli, *Phys. Rev. B*, 2008, **77**, 075133.
12. C. Wu, D. A. Thomas, Z. B. Lin and L. V. Zhigilei, *Appl. Phys. A*, 2011, **104**, 781-792.
13. R. H. M. Groeneveld, R. Sprik and A. Lagendijk, *Phys. Rev. B*, 1995, **51**, 11433-11445.
14. K. C. Mills, B. J. Monaghan and B. J. Keene, *Int. Mater. Rev.*, 1996, **41**, 209-242.
15. S. M. Foiles, M. I. Baskes and M. S. Daw, *Phys. Rev. B*, 1986, **33**, 7983-7991.
16. A. F. Voter and S. P. Chen, *Mat. Res. Soc. Symp. Proc.*, 1986, **82**, 175-180.
17. S. M. Foiles and J. B. Adams, *Phys. Rev. B*, 1989, **40**, 5909-5915.
18. W. M. Haynes, *Handbook of chemistry and physics*, CRC Press, Boca Raton, Florence, 97th edn., 2016.
19. E. T. Karim, M. Shugaev, C. P. Wu, Z. B. Lin, R. F. Hainsey and L. V. Zhigilei, *J. Appl. Phys.*, 2014, **115**, 183501.
20. B. J. Garrison, T. E. Itina and L. V. Zhigilei, *Phys. Rev. E*, 2003, **68**, 041501.
21. A. Miotello and R. Kelly, *Appl. Phys. A*, 1999, **69**, S67-S73.
22. N. M. Bulgakova and A. V. Bulgakov, *Appl. Phys. A*, 2001, **73**, 199-208.
23. R. W. Ohse and H. v. Tippelskirch, *High Temp-High Press*, 1977, **9**, 367-385.
24. L. V. Zhigilei, E. Leveugle, B. J. Garrison, Y. G. Yingling and M. I. Zeifman, *Chem. Rev.*, 2003, **103**, 321-347.
25. L. V. Zhigilei, P. B. S. Kodali and B. J. Garrison, *J. Phys. Chem. B*, 1997, **101**, 2028-2037.

26. D. J. Phares and A. R. Srinivasa, *J. Phys. Chem. A*, 2004, **108**, 6100-6108.

27. W. M. Jacobs, D. A. Nicholson, H. Zemer, A. N. Volkov and L. V. Zhigilei, *Phys. Rev. B*, 2012, **86**, 165414.

28. M. Tabetah, A. Matei, C. Constantinescu, N. P. Mortensen, M. Dinescu, J. Schou and L. V. Zhigilei, *J. Phys. Chem. B*, 2014, **118**, 13290-13299.

29. C.-Y. Shih, C. P. Wu, M. V. Shugaev and L. V. Zhigilei, *J. Colloid Interface Sci.*, 2017, **489**, 3-17.

30. A. Michaelides, V. A. Ranea, P. L. de Andres and D. A. King, *Phys. Rev. Lett.*, 2003, **90**, 216102.

31. J. Ren and S. Meng, *Phys. Rev. B*, 2008, **77**, 054110.

32. Z. E. Hughes, L. B. Wright and T. R. Walsh, *Langmuir*, 2013, **29**, 13217-13229.

33. A. Michaelides, A. Alavi and D. A. King, *Phys. Rev. B*, 2004, **69**, 113404.

34. Y. G. Yingling, L. V. Zhigilei and B. J. Garrison, *J. Photochem. Photobiol. A*, 2001, **145**, 173-181.

35. Y. G. Yingling, L. V. Zhigilei and B. J. Garrison, *Nucl. Instrum Meth. B*, 2001, **180**, 171-175.

36. J. Lam, D. Amans, C. Dujardin, G. Ledoux and A.-R. Allouche, *J. Phys. Chem. A*, 2015, **119**, 8944-8949.

37. X. Sedao, M. V. Shugaev, C. Wu, T. Douillard, C. Esnouf, C. Maurice, S. Reynaud, F. Pigeon, F. Garrelie, L. V. Zhigilei and J.-P. Colombier, *ACS Nano*, 2016, **10**, 6995-7007.

38. Yu. V. Petrov, N. A. Inogamov, S. I. Anisimov, K. P. Migdal, V. A. Khokhlov and K. V. Khishchenko, *J. Phys: Conf. Ser.*, 2015, **653**, 012087.

39. Y. Ren, J. K. Chen, Y. Zhang and J. Huang, *Appl. Phys. Lett.*, 2011, **98**, 191105.

40. X. Y. Wang, D. M. Riffe, Y. S. Lee and M. C. Downer, *Phys. Rev. B*, 1994, **50**, 8016-8019.

41. E. L. Gurevich, Y. Levy, S. V. Gurevich and N. M. Bulgakova, *Phys. Rev. B*, 2017, **95**, 054305.

42. E. D. Palik, *Handbook of optical constants of solids*, Academic Press, 1998.

43. J. Hohlfeld, S. S. Wellershoff, J. Güdde, U. Conrad, V. Jähnke and E. Matthias, *Chem. Phys.*, 2000, **251**, 237-258.
